# Difficult biliary cannulation during endoscopic retrograde cholangiopancreatography for distal malignant biliary obstruction caused by pancreatic cancer: An observational study

**DOI:** 10.1002/deo2.70092

**Published:** 2025-03-04

**Authors:** Jun Noda, Yuichi Takano, Naoki Tamai, Masataka Yamawaki, Tetsushi Azami, Fumitaka Niiya, Naotaka Maruoka, Masatsugu Nagahama

**Affiliations:** ^1^ Department of Internal Medicine, Division of Gastroenterology Showa University Fujigaoka Hospital Kanagawa Japan

**Keywords:** bile ducts, cholangiopancreatography, drainage, pancreatic carcinoma, retrospective studies

## Abstract

**Objectives:**

Distal malignant biliary obstruction (DMBO) caused by pancreatic cancer often complicates endoscopic retrograde cholangiopancreatography (ERCP), particularly biliary cannulation. While various factors influencing difficult biliary cannulation (DBC) have been studied, data specific to pancreatic cancer‐related distant malignant biliary obstruction#x000A0;remains limited. This study identifies factors affecting ERCP success in this patient population to improve clinical outcomes.

**Methods:**

We retrospectively analyzed 119 ERCP procedures for distant malignant biliary obstruction owing to pancreatic cancer with naïve papilla at Showa University Fujigaoka Hospital (January 2020–September 2024). Patient characteristics, duodenal invasion, ampullary bile duct status, papillary morphology, trainee involvement, and adverse events were evaluated. Multivariate analysis identified predictive factors of DBC.

**Results:**

After excluding 17 ERCP failures, 102 patients were analyzed and categorized into non‐DBC (*n* = 40) and DBC (*n* = 62) groups. The DBC incidence rate was 60.8%. The absence of the ampullary bile duct (odds ratio [OR]: 2.58, 95% confidence interval [CI]: 1.02–6.51; *p* = 0.04) and the macroscopic appearance of type III papillary morphology (enlarged/protruding; OR: 3.32; 95% CI: 1.07–10.30; *p* = 0.04) were significantly associated with DBC. Adverse events were slightly more frequent in the DBC group; however, this difference was not statistically significant. Alternative cannulation was performed more often in patients without the ampullary bile duct; however, no difference in adverse events was observed.

**Conclusions:**

The absence of the ampullary bile duct and type III papillary morphology are anatomical risk factors for DBC during ERCP for patients with pancreatic cancer. Early consideration of alternative cannulation techniques or biliary drainage methods may be necessary for such patients.

## INTRODUCTION

Endoscopic retrograde cholangiopancreatography (ERCP) is a complex therapeutic endoscopy procedure, with deep biliary cannulation being a critical initial step.^1^ However, difficult biliary cannulation (DBC) occurs in some patients, posing a significant challenge.

The European Society of Gastrointestinal Endoscopy defines DBC based on the “5‐5‐1 criteria,” which include five or more cannulation attempts over 5 min to achieve cannulation after contacting the papilla and multiple inadvertent pancreatic duct cannulations or opacifications.^2^ The reported incidence of DBC ranges from 5% to 49%, with an average of approximately 20%.^3^, ^4^

Several factors have been identified as potential predictors of DBC, including distal malignant biliary obstruction (DMBO) caused by pancreatic cancer, the experience of the endoscopist, and papillary anatomy.^5^ ^–‐^ ^13^ DMBO owing to pancreatic cancer is a common indication for ERCP,^14^ yet studies specifically addressing factors associated with DBC in this patient group remain limited. Compared to bile duct and ampullary cancers, pancreatic cancer is linked to a higher risk of DBC^11^ and longer cannulation times.^15^

Pancreatic tumors can distort the duodenal and papillary anatomy through compression or direct invasion, further complicating cannulation.^16^ Direct duodenum invasion is a known risk factor for ERCP failure.^11^ However, even in cases where the duodenum and papilla appear normal, the absence of a visible bile duct opening at the papilla may contribute to DBC, possibly due to the lack of an ampullary bile duct.

Identifying high‐risk patients enables endoscopists to select the most appropriate cannulation techniques, improving procedural success. Therefore, this study identified the predictors of DBC in patients with DMBO caused by pancreatic cancer, incorporating the absence of a papillary bile duct alongside previously reported risk factors, such as papillary morphology and trainee involvement.

## METHODS

### Patients

We retrospectively analyzed ERCP data from Showa University Fujigaoka Hospital between January 1, 2020, and September 30, 2024, to identify factors associated with DBC in patients with DMBO owing to pancreatic cancer. Inclusion criteria were patients with DMBO caused by pancreatic cancer and a naïve papilla for ERCP. Exclusion criteria included surgically altered anatomy, ERCP failure due to duodenal obstruction, and insufficient data.

### ERCP procedure

The patient was sedated using 35 mg pethidine hydrochloride and 2–5 mg midazolam, followed by insertion of a standard duodenoscope (JF260 V, TJF‐Q260 V, or TJF‐290 V; Olympus Medical Systems). A tapered catheter (PR‐V614 M; Olympus) with an angled‐tip 0.025‐inch guide wire (Visiglide2; Olympus) was used. If conventional bile duct cannulation (contrast‐ and wire‐guided) failed, and the guidewire entered the pancreatic duct, the double‐guidewire technique (DGW) or transpancreatic biliary sphincterotomy (TPBS) was attempted using a sphincterotome (CleverCut3 V; Olympus). If the pancreatic duct could not be accessed, a direct precutting technique was applied at the endoscopist's discretion of the endoscopist. Prophylactic pancreatic stent placement was optional, and rectal diclofenac, along with large‐volume fluid administration, was used to prevent post‐ERCP pancreatitis (PEP). If intubation was difficult, we promptly attempted alternative cannulation methods, with trainees performing TPBS or direct precutting. If successful, trainees proceeded under expert supervision; otherwise, an expert took over after 15–20 min. Endoscopic sphincterotomy was performed unless contraindicated by bleeding risk or anticoagulant use, in which case endoscopic papillary balloon dilation was performed with an 8‐mm balloon. Plastic stents (7‐Fr or 8.5‐Fr, straight or pigtail) or fully covered metal stents (8 or 10 mm in diameter and 6 or 8 cm in length) were used as needed.

### Definitions and outcomes

DBC was defined by the 5‐5‐1 criteria of the European Society of Gastrointestingal Endoscopy: cannulation time ≥5 min; ≥5 catheter insertion attempts; or ≥1 pancreatic duct cannulation.^2^ Patients who were handed over from the trainee to the expert and then successfully cannulated shortly thereafter were classified into the non‐DBC group. Successful biliary cannulation was defined as deep cannulation of the bile duct, with cannulation time measured from scope insertion to deep cannulation using timestamps from endoscopic and fluoroscopic images. Cholangitis was defined according to the 2018 Tokyo Guidelines proposed by the Japan Society of Hepato‐Biliary‐Pancreatic Surgery. ^17^ Clinical stage was determined using the 8th edition of the TNM classification by the Japan Pancreas Society.^18^

**FIGURE 1:**
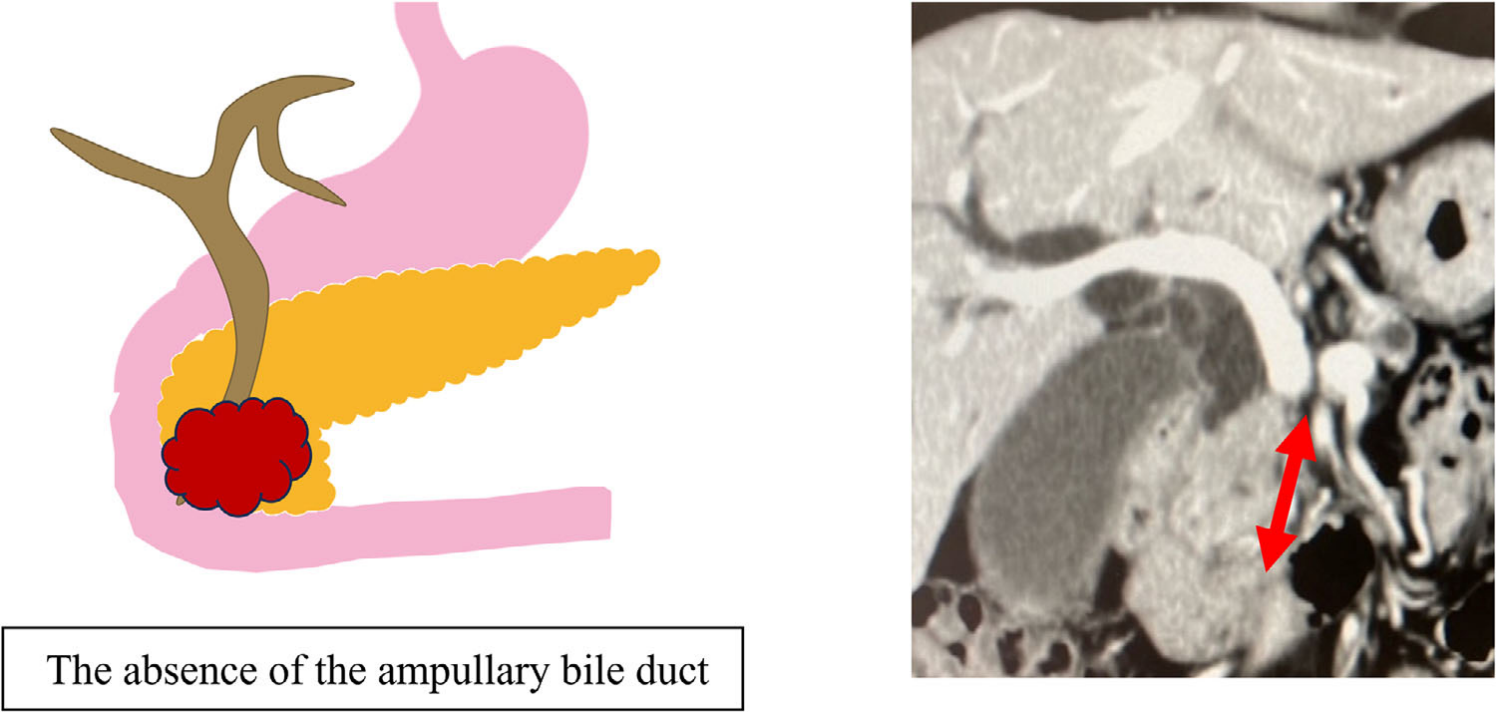
Schematic diagram and computed tomography image showing the absence of the ampullary bile duct (red arrow).

The absence of the ampullary bile duct was defined as a continuous stricture ≥5 mm from the ampullary region, observed on preoperative computed tomography (Figure 1). The duodenal invasion was defined as a tumor invasion observed during endoscopy.

**FIGURE 2:**
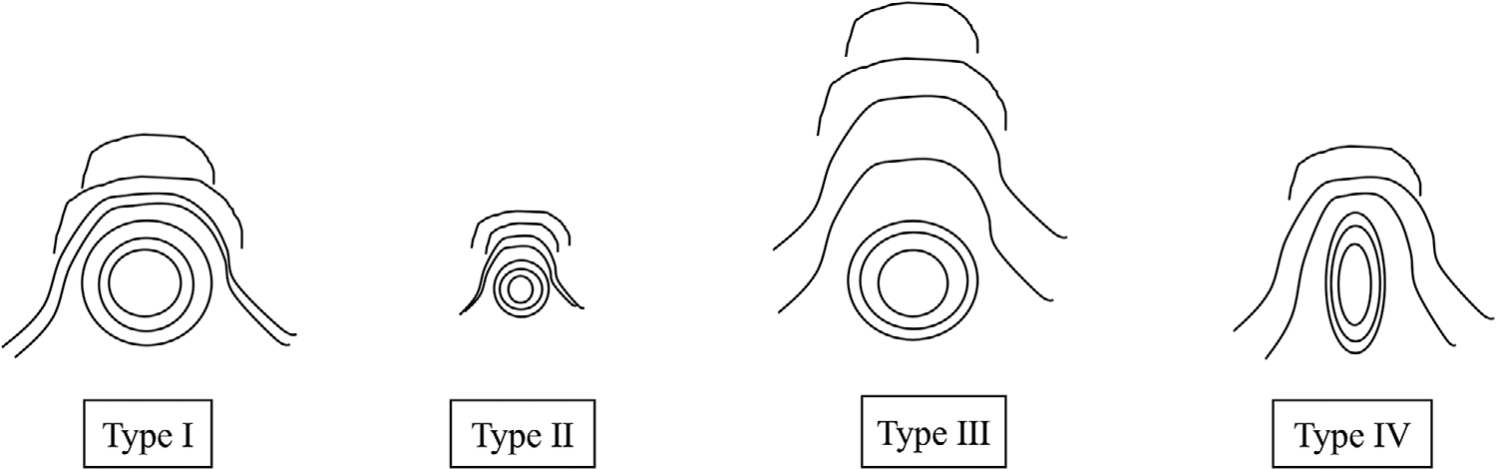
Macroscopic ampullary appearance types I–IV according to Haraldsson's classification.

Papillary morphology was classified according to Haraldsson's category: type I, normal; type II, small; type III, enlarged/protruding; and type IV, ridged (Figure 2).^19^ Type III papilla classification followed Watanabe et al., measuring the length of the oral protrusion using the marker at the ERCP catheter tip. Periampullary diverticula were documented and classified using Boix's categories (types I, II, and III).^20^

Experts were defined as physicians with ≥400 ERCP procedures; trainees had performed fewer than 400.^21^ At our institution, trainees are not beginners in initial ERCP for DMBO, having completed >50 procedures. Three experts and five trainees participated in this study.

Adverse events (AEs) were defined and graded according to the American Society for Gastrointestinal Endoscopy severity system.^22^ Pancreatitis was diagnosed with abdominal pain and serum amylase ≥3 times the upper normal limit. Bleeding was confirmed by visible evidence and a hemoglobin decrease of <3 g/dL. Perforation was confirmed by the presence of air or luminal contents outside the gastrointestinal tract. Outcomes were defined as differences in predictive factors for DBC, AEs, and alternative cannulation between the non‐DBC and DBC groups.

### Statistical analysis

Continuous variables were presented as medians with interquartile ranges and analyzed using the Mann–Whitney U test. Categorical variables were expressed as proportions and compared using Fisher's exact test. A multivariate logistic regression analysis assessed factors associated with DBC, including duodenal invasion, absence of the ampullary bile duct, trainee involvement, and papillary morphology. *p* < 0.05 was considered statistically significant. All analyses were conducted using R version 3.4.1 (The R Foundation for Statistical Computing).

## RESULTS

### Patient characteristics

**FIGURE 3:**
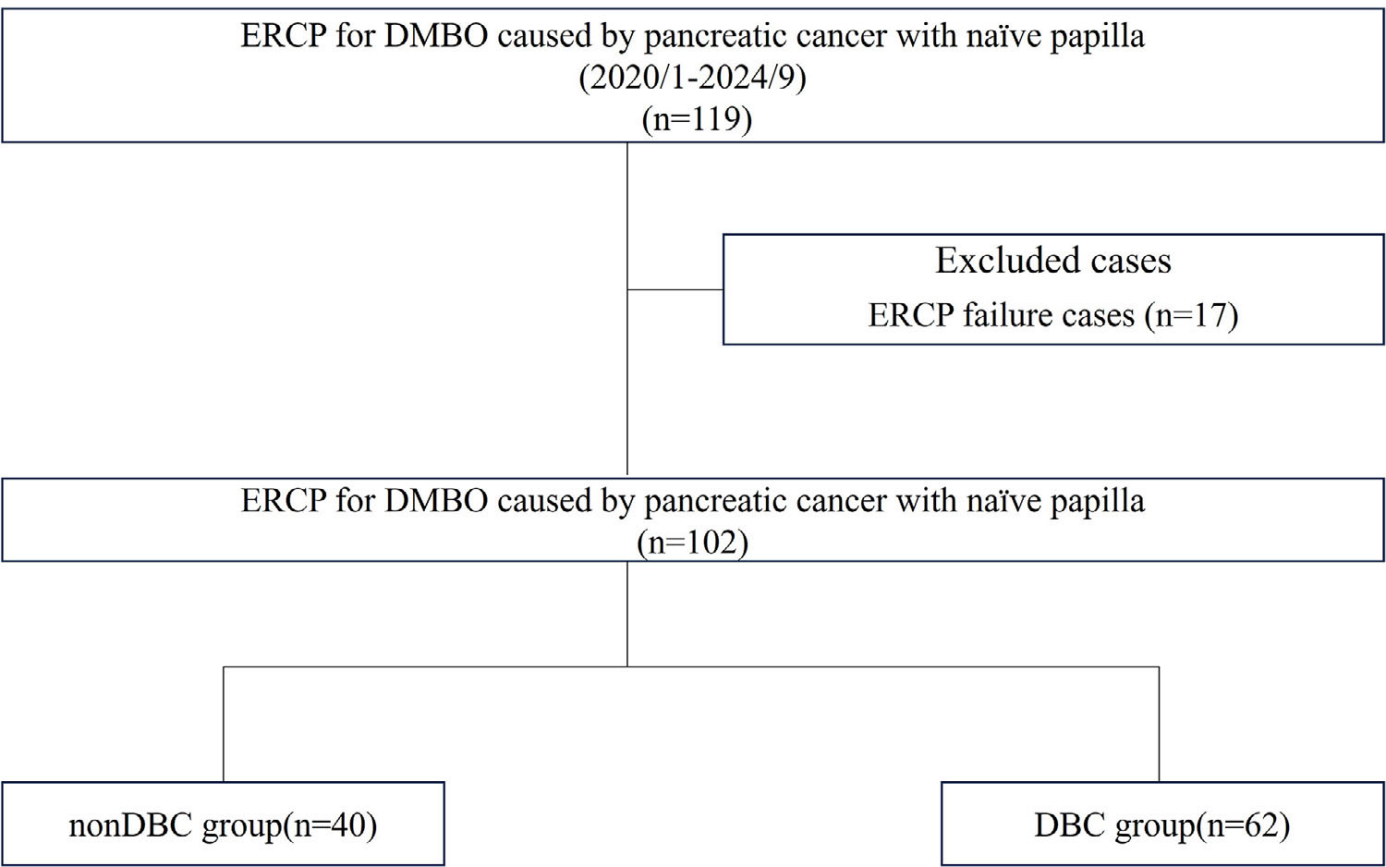
Patient flow chart. DBC, difficult biliary cannulation; DMBO, distal malignant biliary obstruction; ERCP, endoscopic retrograde cholangiopancreatography.

Between January 1, 2020, and September 30, 2024, 119 patients with DMBO due to pancreatic cancer and naïve papilla undergoing ERCP were screened. Seventeen patients were excluded due to ERCP failure caused by duodenal obstruction (13 switched to endoscopic ultrasound‐guided biliary drainage [EUS‐BD], one to percutaneous transhepatic gallbladder drainage, and three discontinued due to worsening condition). Therefore, 102 patients were included in the analysis: 40 and 62 in the non‐DBC and DBC groups, respectively (Figure 3).

Table 1 summarizes the patient characteristics. The median age in the non‐DBC group was 73.5 years, and in the DBC group, it was 76.5 years. The proportion of females was similar in both groups: 57.5% in the non‐DBC group and 53.2% in the DBC group. The median pre‐ERCP total bilirubin levels were 5.15 and 6.5 mg/dL in the non‐DBC and DBC groups, respectively; this difference was not statistically significant. The median bile duct diameters were 15 mm and 16 mm in the non‐DBC and DBC groups, respectively. The median lengths of the bile duct stricture measured using fluoroscopy were 25.5 and 23.5 mm in the non‐DBC and DBC groups, respectively.

**TABLE 1:** Baseline parameters, endoscopic procedure characteristics, and clinical outcomes of the patients.

	Non‐DBC group *n* = 40	DBC group *n* = 62	*p*‐value
Age, median [IQR], (years)	73.5 (43–96)	76.5 (34–93)	0.147
Female sex, *n* (%)	23 (57.5)	33 (53.2)	0.689
Total bilirubin before the ERCP procedure, median [IQR], (mg/dL)	5.15 (0.3–27)	6.5 (0.6–26)	0.671
Presence of cholangitis, *n* (%)	4 (10.0)	5 (8.1)	0.735
Bile duct diameter, median [IQR], (mm)	15 (9–32)	16 (7–29)	0.404
Bile duct stricture length on CT, median [IQR], (mm)	25.5 (9–45)	23.5 (8–45)	0.755
Clinical stage, *n* (%)			0.423
Stage I	0 (0)	2 (3.2)	
Stage II	15 (37.5)	15 (24.2)	
Stage III	11 (27.5)	22 (35.5)	
Stage IV	14 (35.0)	23 (37.1)	
Treatment policy, *n* (%)			0.506
Surgery	8 (20.0)	10 (16.1)	
Chemotherapy	10 (25.0)	22 (35.5)	
Best supportive care	22 (55.0)	30 (48.4)	
Cannulation time, median [IQR], (min)	4.5 (1–5)	21.5 (4–54)	<0.001*
Cannulation method, *n* (%)			<0.001*
Normal	40 (100)	9 (14.5)	
Double‐guidewire	0 (0)	33 (53.2)	
TPBS	0 (0)	11 (17.7)	
Direct precutting	0 (0)	9 (14.5)	
Papilla treatment, *n* (%)			0.886
None	2 (5.70)	2 (3.2)	
EST	36 (90.0)	56 (90.3)	
EPBD	2 (5.0)	4 (6.0)	
Biliary stenting, *n* (%)			1.000
Plastic stent	31 (77.5)	49 (79.0)	
Metallic stent	9 (22.5)	13 (21.0)	
Adverse events, *n* (%)			0.126
Pancreatitis (mild)	1 (2.5)	7 (11.3)	
Bleeding	0 (0)	3 (4.8)	
Perforation	0 (0)	0 (0)	

Abbreviations: CT, computed tomography; DBC, difficult biliary cannulation; EPBD, endoscopic papillary balloon dilatation; ERCP, endoscopic retrograde cholangiopancreatography; EST, endoscopic sphincterotomy; IQR interquartile range; TPBS, transpancreatic biliary sphincterotomy.

The biliary cannulation time significantly differed between groups. The median biliary cannulation time in the non‐DBC and DBCs groups was 4.5 and 21.5 min, respectively. All patients in the non‐DBC group underwent conventional cannulation, whereas the DBC group included patients with a cannulation time of ≥5 min using the conventional technique (*n* = 9, 14.6%), those who underwent only the double‐guidewire technique (*n* = 33, 53.2%), those who had the double‐guidewire technique followed by TPBS (*n* = 11, 17.7%), and those requiring direct precutting after failed double‐guidewire attempts (*n* = 9, 14.5%).

No significant differences in AE rates were observed between the groups, although the DBC group had a higher AE rate. Mild pancreatitis occurred in one (2.5%) patient in the non‐DBC group and seven (11.3%) in the DBC group, but this difference was not statistically significant. Bleeding was observed in three (4.8%) patients in the DBC group, but this finding was also not statistically significant.

### Factors predictive of DBC

Table 2 presents the prevalence of factors predictive of DBC, along with the results of the univariate and multivariate analyses. Regarding papillary morphology, 12.5% and 30.6% of patients in the non‐DBC and DBC groups, respectively, were classified as type III. Periampullary diverticula were observed in 15.0% and 4.8% of type I cases in the non‐DBC and DBC groups, respectively, 0.0% and 1.6% of type II cases, and 0.0% and 3.2% of type III cases. The duodenal invasion was present in 12.5% and 17.7% of patients in the non‐DBC and DBC groups, respectively. Trainees were involved in 67.5% and 71.0% of cases in the non‐DBC and DBC groups, respectively. The absence of the ampullary bile duct was observed in 27.5% and 51.6% of patients in the non‐DBC and DBC groups, respectively.

**TABLE 2:** Univariate and multivariate analysis results of the predictive factors for difficult biliary cannulation.

	Difficult biliary cannulation		Univariate analysis		Multivariate analysis	
	Yes (*n* = 62)	No (*n* = 40)	Unadjusted odds ratio (95% CI)	*p*‐value	Adjusted odds ratio (95% CI)	*p*‐value
Macroscopic appearance of the ampulla, *n* (%)						
Type I	40 (64.5)	30 (75.0)	Ref			
Type II	3 (4.8)	4 (10.0)	0.461 (0.064–2.897)	0.428		
Type III	19 (30.6)	5 (12.5)	3.061 (0.975–11.57)	0.054	3.32 (1.07–10.30)	0.04*
Type IV	0 (0.0)	1 (2.5)	0 (0.000–25.16)	0.392		
Periampullary diverticulum, *n* (%)						
No diverticulum	56 (90.3)	34 (85.0)	Ref			
Type I	3 (4.8)	6 (15.0)	0.292 (0.044–1.471)	0.149	0.30 (0.07–1.37)	0.12
Type II	1 (1.6)	0 (0.0)	Inf (0.016–Inf)	1		
Type III	2 (3.2)	0 (0.0)	Inf (0.121–Inf)	0.519		
Duodenal invasion, *n* (%)						
No	51 (82.3)	35 (87.5)	Ref			
Yes	11 (17.7)	5 (12.5)	1.503 (0.434–6.022)	0.583	1.13 (0.32–3.97)	0.85
Trainee involvement, *n* (%)						
No	18 (29.0)	13 (32.5)	Ref			
Yes	44 (71.0)	27 (67.5)	1.175 (0.451–3.012)	0.826	1.41 (0.55–3.57)	0.47
The absence of the ampullary bile duct, *n* (%)						
No	30 (28.4)	29 (72.5)	Ref			
Yes	32 (51.6)	11 (27.5)	2.783 (1.113–7.334)	0.023*	2.58 (1.02–6.51)	0.04*

95% CI, 95% Confidence Interval; Ref, reference.

In the univariate analysis, the absence of the ampullary bile duct was significantly associated with DBC (odds ratio [OR]: 2.783; 95% confidence interval [95% CI]: 1.113–7.334; *p* = 0.023).

In addition to all variables with *p* < 0.2 in univariate analysis, trainee involvement and duodenal invasion were included in the multivariate analysis because they were considered significant variables clinically associated with DBC. In this analysis, type III (enlarged/protruding) papillary morphology, type I periampullary diverticulum, duodenal invasion, trainee involvement, and the absence of the ampullary bile duct were identified as factors related to DBC. Type III (enlarged/protruding) papillary morphology was significantly associated with DBC (OR: 3.32; 95% CI: 1.07–10.30; *p* = 0.04). The absence of the ampullary bile duct was also significantly associated with DBC (OR: 2.58; (95% CI: 1.02–6.51; *p* = 0.04).

The characteristics of patients without the ampullary bile duct are shown in Table 3. Compared to those with the ampullary bile duct, patients without it had a significantly higher DBC rate (74.4% vs. 50.8%) and longer cannulation times (19 min vs. 10 min). Additionally, alternative cannulation was more common in the group without the ampullary bile duct (61.3% vs. 41.8%). No significant difference in the AE rates was observed between the two groups (9.4% vs. 11.9%). The pancreatitis rate was lower in patients without the ampullary bile duct compared to those with it (4.7% vs. 10.2%).

**TABLE 3:** Patient characteristics regarding the absence of the ampullary bile duct.

	Absence of the ampullary bile duct		
	Yes (*n* = 43)	No (*n* = 59)	*p*‐value
Difficult biliary cannulation, *n* (%)			
Yes	32 (74.4)	30 (50.8)	0.023*
No	11 (25.6)	29 (49.2)	
Cannulation time, median [IQR], (min)	19.00 (2–54)	10.00 (2–54)	0.01*
Bile duct stricture length on CT, median [IQR], (mm)	23.00 (1–45)	25.00 (8–42)	0.927
Cannulation method, *n* (%)			0.021*
Normal	16 (37.2)	33 (55.9)	
Double‐guidewire	13 (30.2)	20 (33.9)	
TPBS	9 (20.9)	2 (3.4)	
Direct precutting	5 (11.6)	4 (6.8)	
Adverse event, *n* (%)			
Pancreatitis	2 (4.7)	6 (10.2)	0.462
Bleeding	2 (4.7)	1 (1.7)	0.572
Perforation	0 (0.0)	0 (0.0)	

Abbreviations: CT, computed tomography; IQR, interquartile range; TPBS, transpancreatic biliary sphincterotomy.

## DISCUSSION

This retrospective study was the first to investigate factors associated with DBC during ERCP for DMBO caused by pancreatic cancer. Successful bile duct cannulation is the most critical step in the ERCP procedure, and identifying predictive factors for DBC can benefit both patients with pancreatic cancer and endoscopists.

The incidence of DBC in DMBO caused by pancreatic cancer was 60.8%, which aligns with the 56.4% reported in the literature.^11^ This high incidence highlights the significant risk that DMBO presents to successful biliary cannulation during ERCP, which is essential for guiding treatment decisions and discussing risks with patients. Multivariate analysis identified the absence of the ampullary bile duct and type III (enlarged/protruding) papillary morphology as primary factors influencing biliary cannulation. These findings highlight the important roles of papillary morphology and the status of the ampullary bile duct in successful bile duct cannulation.

ERCP has been extensively studied as a treatment for DMBO, with reported success and cannulation rates.^6^, ^9^, ^10^, ^11^, ^13^, ^14^, ^15^ DMBO caused by pancreatic cancer often disrupts the anatomy of the duodenum and papilla, complicating endoscopic maneuvering.^16^ In patients with pancreatic cancer, compromised biliary duct and changes in the duodenal mucosa can lead to AEs such as papillary edema and bleeding, further complicating the cannulation process.^23^ Fugazza et al. identified DMBO caused by pancreatic cancer as a risk factor for DBC, suggesting that tumor invasion into the ampulla is associated with DBC, while invasion into the duodenum is linked to ERCP failure.^11^ In our study, cases of complete duodenal obstruction caused by tumor invasion were excluded, and duodenal invasion did not affect the predictive factors of DBC. While duodenal invasion, excluding papilla invasion, may impact endoscope mobility, it did not determine DBC. Future case accumulation may clarify the trends of DBC and ERCP failure by examining the degree and site of duodenal invasion. Even when tumor invasion is not visible during an endoscopy, the absence of the ampullary bile duct due to tumor involvement may prevent guide‐wire advancement along the correct route. In such cases, the guide wire may create a fistula or result in an intimal injection of contrast, leading to further swelling of the papilla and complicating bile duct intubation. Our study demonstrates that even in the absence of visible invasion, a tumor‐induced stricture that leads to the absence of the ampullary bile duct is a significant risk factor for DBC.

Herein, type III (enlarged/protruding) papillary morphology was considered a risk factor for DBC; however, a long, enlarged, or protruding papilla, such as type III, has been reported as a risk factor for DBC and is not limited to DMBO.^19^, ^24^ In such cases, correctly positioning the papilla in front of the bile duct axis is challenging. Moreover, switching from trainee to expert promptly or, if a wire can be inserted in the pancreatic duct, immediately consider alternative cannulation techniques.

Although previous studies have identified factors associated with DBC, such as trainee involvement^5^, ^12^, ^13^ and the presence of periampullary diverticula, ^8^, ^10^, ^12^ these were not significant risk factors in our analysis. While trainee involvement can extend procedural time and increase the likelihood of AEs after ERCP,^25^, ^26^ evidence suggests it does not always increase AE rates.^27^ In our study, trainee involvement was not a risk factor for DBC, possibly because trainees had prior ERCP experience and expert guidance was consistently available at our facility.

The overall AE rate in our cohort was 10.8% (11/102), comparable to incidence rates reported for all ERCP indications.^28^ DBC increases the risk of AEs, as prolonged procedures increase the likelihood of PEP.^2^, ^29^ Current literature supports TPBS and direct precutting as effective salvage techniques for DBC.^30^, ^31^, ^32^, ^33^, ^34^ Implementing an early alternative technique, rather than delaying after prolonged cannulation attempts, can improve safety and efficacy.^31^ In our study, 85.4% (53/62) of patients in the DBC group underwent alternative cannulation methods, including TPBS or direct precutting. Prompt use of these techniques in patients with multiple DBC risk factors may improve procedural efficiency and reduce AEs. Additionally, DBC was significantly more common among patients without the ampullary bile duct, most of whom required alternative cannulation methods; however, these patients experienced fewer AEs (Table 3). Patients without an ampullary bile duct had a lower rate of PEP compared to those with one. The reason for this remains unclear. One possible explanation is that when the ampullary bile duct is absent, the risk of PEP increases due to prolonged bile duct cannulation time and obstruction of the main pancreatic duct by a tumor near the papilla. However, the lower PEP rate may be attributed to the timing of the switch to an alternative cannulation method, which may explain why the incidence of PEP was lower in patients without an ampullary bile duct.

The effectiveness of EUS‐BD for DMBO has been documented, with studies comparing ERCP and EUS‐BD reporting fewer AEs with EUS‐BD.^35^ Therefore, from a trainee education perspective, early transition to an alternative cannulation method is considered acceptable. However, when DBC is anticipated before ERCP, early conversion to EUS‐BD is preferable. as it may reduce the risk of AEs.

This study highlights the impact of anatomical factors on ERCP outcomes. Specifically, type III (enlarged/protruding) papillary morphology and the absence of the ampullary bile duct significantly influence DBC success. Therefore, these anatomical features should be carefully considered. While papillary morphology can only be assessed endoscopically, these findings underscore the importance of using computed tomography to evaluate the presence of the ampullary bile duct before ERCP in patients with DMBO caused by pancreatic cancer.

This study has some limitations. As a retrospective study, it may be subject to selection and data collection biases. While this study underscores the influence of the absence of the ampullary bile duct on ERCP success, the specific anatomical factors influencing DBC remain unclear. Future research is needed to clarify how specific aspects of the papillary morphology and anatomical structure contribute to DBC.

In summary, this study identifies type III (enlarged/protruding) papillary morphology and the absence of the ampullary bile duct as key factors contributing to DBC during ERCP in patients with DMBO caused by pancreatic cancer. Recognizing the absence of the ampullary bile duct before ERCP and promptly employing alternative cannulation techniques or converting to EUS‐BD could improve patient safety.

## CONFLICT OF INTEREST STATEMENT

None.

## ETHICS STATEMENT

Approval of the research protocol by an Institutional Reviewer Board: This study was approved by the Institutional Review Board of the hospital on April 21, 2024.

## PATIENT CONSENT STATEMENT

The requirement for written informed consent from the patients was waived due to the retrospective nature of the study.

## CLINICAL TRIAL REGISTRATION

N/A.
